# Emotion regulation resource profiles among employees: mental health and work outcomes

**DOI:** 10.3389/fpsyt.2026.1873483

**Published:** 2026-07-30

**Authors:** Jinrui Tian, Ping Jia, Ronghua Zhang, Zhe Jin

**Affiliations:** 1 Institute of Developmental and Educational Psychology, School of Marxism, Wuhan University, Wuhan, China; 2 Shaanxi Youth Vocational College, Xi’an, China; 3 Department of Ideological and Political Education, School of Marxism, Wuhan University, Wuhan, China

**Keywords:** emotion regulation resources, emotional labor, employee mental health, latent profile analysis, psychological capital, work engagement

## Abstract

Workplace stress is closely related to employes’ mental health and work functioning, but most research has examined emotional labor, emotion regulation difficulties, and psychological resources as separate variables. This study addressed this limitation by using a person-centered approach to identify how emotion regulation burdens and resources are configured within employees and how these configurations relate to mental health and work outcomes. A cross-sectional questionnaire survey was conducted with 1,552 Chinese employees who attended workplace mental health and emotion management seminars. Latent profile analysis was performed using six indicators: surface acting, deep acting, difficulties in emotion regulation, resilience, self-efficacy, and optimism. Profile differences in depression, insomnia, psychological well-being, work engagement, and innovative work behavior were assessed, and demographic correlates of profile membership were examined using multinomial logistic regression. Four profiles were identified: high-risk dysregulated, vulnerable low-resource, normative moderate, and high-resource adaptive. The profiles showed a clear resource-risk gradient. Employees in the high-risk dysregulated profile reported the highest depression and insomnia and the lowest positive work outcomes, whereas those in the high-resource adaptive profile showed the most favorable pattern. Gender, education, and managerial position were significant correlates of profile membership. The study contributes by showing that workplace emotion regulation risk is not captured by single indicators alone, but by meaningful configurations of demands, difficulties, and resources. The findings may inform workplace mental health risk identification, targeted emotion regulation training, and resource-building interventions.

## 1 Introduction

Workplace stress is a major concern for employee mental health and organizational functioning. International and occupational health reports link mental health problems at work with poorer employment outcomes, absenteeism, presenteeism, and productivity loss (1–3). Empirical reviews and recent occupational studies further show that work-related strain is associated with depression, insomnia, burnout, and impaired functioning (4–6).

Emotion regulation is central to this issue because many employees must manage both internal feelings and outward emotional displays in response to job demands. Existing research has linked emotional labor and emotion regulation difficulties to poorer mental health, whereas resilience, self-efficacy, and optimism are generally discussed as protective resources (7–10).

However, much of this evidence remains variable-centered. Such approaches clarify average associations among variables, but they do not show whether employees combine emotional demands, regulation difficulties, and psychological resources in distinct within-person patterns. This gap is important because similar levels of one burden may have different meanings depending on the broader resource context in which that burden is embedded. To address this gap, the present study used latent profile analysis to identify configurations of emotion regulation burdens and psychological resources among Chinese employees. The study then examined whether these profiles differed in mental health and work outcomes and whether demographic characteristics were associated with profile membership. This person-centered approach was intended to provide a clearer account of workplace emotion regulation risk and resource protection.

## 2 Literature review

### 2.1 Workplace stress and emotion regulation resources

Workplace stress is commonly understood as a process in which individuals appraise job demands and evaluate whether they have sufficient resources to cope with them (11). The transactional model of stress emphasizes appraisal and coping, whereas the process model of emotion regulation explains how individuals modify emotional experience and expression across different stages of an emotional episode (12, 13). In the workplace, these processes are not purely individual. They are embedded in organizational demands, display rules, and available personal and social resources.

Emotional labor is a central emotional demand in many work roles because employees are often required to regulate their feelings and expressions as part of their job performance (14). It refers to the regulation of internal feelings and outward expressions to meet work-related display rules. Surface acting involves suppressing or faking emotions, whereas deep acting involves trying to change inner emotional experience through reappraisal or empathy (15). Surface acting is generally more depleting and has strong links with burnout and other negative outcomes (7, 16). Studies also connect emotional labor with depressive symptoms and sleep problems in healthcare workers (17, 18). More recent evidence identifies burnout as a key pathway linking emotional labor to adverse outcomes (19, 20). By contrast, deep acting is usually less harmful and may be associated with job satisfaction and performance when employees have sufficient resources (21).

Difficulties in emotion regulation refer to limitations in emotional awareness, acceptance, impulse control, goal-directed behavior, and access to effective regulation strategies (22). These difficulties represent an internal resource deficit. They are consistently associated with depression and insomnia (8, 23). Recent studies also link emotion dysregulation to insomnia-related symptoms and psychiatric comorbidity (24, 25). In the workplace, such difficulties may mark a lower capacity for emotional recovery under job demands.

Resilience and psychological capital are widely viewed as protective personal resources in work and health contexts (26, 27). Resilience refers to the capacity to maintain or regain functioning under adversity. Psychological capital reflects a positive psychological state involving self-efficacy, hope, optimism, and resilience (28). In work settings, these resources are linked with lower distress, stronger engagement, and better performance (29, 30). Recent organizational research also shows that psychological capital and work engagement are positively related to job performance (31). The present study therefore treats emotional labor, emotion regulation difficulties, resilience, self-efficacy, and optimism as interrelated components of emotion regulation resource configurations.

### 2.2 Emotion regulation resources, stress, and health and work outcomes

Workplace stress research increasingly emphasizes the need to examine both negative health outcomes and positive indicators of adaptation (1, 32, 33). In this study, depression and insomnia were selected as risk outcomes because they are common consequences of chronic stress and are highly relevant to workplace functioning. Depression reflects affective and motivational impairment, while insomnia indicates difficulty with restorative sleep. Both outcomes are associated with absenteeism, presenteeism, accident risk, and reduced productivity (34, 35).

Psychological well-being, work engagement, and innovative work behavior were included as positive indicators of adaptation because they represent functioning beyond the absence of symptoms (36, 37). Psychological well-being reflects positive functioning, purpose, self-acceptance, environmental mastery, and personal growth (38). Work engagement represents a positive motivational state characterized by vigor, dedication, and absorption (37). Innovative work behavior reflects the generation, promotion, and implementation of new ideas in daily work (39, 40).

The Job Demands-Resources model suggests that high demands and insufficient resources are associated with strain, whereas stronger resources are associated with engagement and performance (41). Employees with high regulation difficulties and frequent surface acting may show poorer outcomes because this pattern combines external emotional demands with internal regulatory burden. Employees with high resilience, self-efficacy, and optimism may show more favorable outcomes because this pattern reflects stronger resource availability. Empirical studies support these links, but most have examined each burden or resource separately rather than as a configuration (42–44).

A profile approach is useful because the meaning of one risk factor may differ depending on the broader configuration of resources (45). For example, high surface acting may correspond to a less favorable pattern when paired with high dysregulation and low resilience. It may correspond to a less severe pattern when paired with stronger psychological resources. This study examines whether such configurations are present in employees and whether they systematically distinguish mental health and work outcomes.

### 2.3 A latent profile perspective: person-centered patterns of emotion regulation resource configurations

Latent profile analysis is a person-centered method that identifies unobserved subgroups based on the joint distribution of multiple continuous indicators (45). Unlike variable-centered approaches, which estimate average relations among variables, person-centered approaches ask whether individuals can be classified into qualitatively meaningful patterns (46). This makes LPA especially suitable for studying the coexistence of emotional demands, regulation difficulties, and psychological resources.

Prior person-centered research shows that coping and resource profiles can reveal patterns that are not visible in single-variable analyses (47). Schlesier and Westphal (47) identified coping profiles among student teachers that differed in perceived stress and stressor appraisal. Klingenberg and Süß (48) identified nurse profiles based on resilience and coping strategies. These profiles differed in job stress and health status.

Person-centered research on emotional labor also demonstrates the value of examining regulatory strategies as profiles rather than as isolated variables (49). Gabriel et al. (49) used LPA to distinguish profiles such as non-actors, surface actors, deep actors, and regulators. Employees who relied heavily on surface acting reported greater emotional exhaustion. Employees who combined more adaptive regulation strategies showed more favorable outcomes. In research on psychological capital, Gao et al. (10) identified enriched, moderate, and depleted profiles among teachers, with the enriched profile reporting higher work engagement.

These studies suggest that emotion regulation and psychological resources should be examined as configurations rather than isolated dimensions (46). However, few studies have simultaneously modeled surface acting, deep acting, emotion regulation difficulties, resilience, self-efficacy, and optimism in a general employee sample. The present study addresses this gap and links the resulting profiles to both health and work outcomes.

### 2.4 Research questions and hypotheses

Consistent with the person-centered logic of the study, the research questions were organized to move from profile identification to outcome differences and demographic correlates. Person-centered studies first establish whether theoretically interpretable profiles can be identified and then examine external outcomes or correlates to clarify the meaning of those profiles (45, 46). On this basis, the study was guided by three research questions.

Research Question 1. What latent profiles of emotion regulation resources can be identified among employees based on surface acting, deep acting, difficulties in emotion regulation, resilience, self-efficacy, and optimism?

Research Question 2. How do these profiles differ in depression, insomnia, psychological well-being, work engagement, and innovative work behavior?

Research Question 3. Are gender, age, education, managerial position, and work experience associated with profile membership?

Hypothesis 1 was formulated as a directional hypothesis because profile enumeration is partly exploratory. Prior person-centered studies show that employees and occupational groups can be classified into meaningful profiles based on emotional labor strategies, coping, resilience, and psychological capital (10, 48, 49). At the same time, surface acting and emotion regulation difficulties are consistently associated with poorer mental health, whereas resilience, self-efficacy, and optimism are linked with more adaptive functioning (7–9). Therefore, the indicators were expected to form interpretable risk and resource configurations rather than a single undifferentiated continuum.

Hypothesis 1. At least four theoretically meaningful profiles were expected to emerge: a high-resource adaptive profile, a high-risk dysregulated profile, a vulnerable low-resource profile, and a moderate profile.

Hypothesis 2 concerned differences in mental health and work outcomes across profiles. The Job Demands-Resources model and Conservation of Resources theory both suggest that resource-poor patterns are associated with strain, whereas resource-rich patterns are associated with engagement and adaptive functioning (41, 50). Prior findings also link emotional labor and dysregulation with burnout, depression, and sleep problems, while psychological capital and related resources are positively associated with well-being, engagement, and performance (17, 21, 31).

Hypothesis 2. The profiles were expected to differ systematically in mental health and work outcomes. Specifically, the high-resource adaptive profile was expected to report the lowest depression and insomnia and the highest psychological well-being, work engagement, and innovative work behavior. In contrast, the high-risk dysregulated profile was expected to show the least favorable outcome pattern. The vulnerable low-resource and moderate profiles were expected to fall between these two groups.

Hypothesis 3 concerned demographic correlates of profile membership. Education may be associated with coping knowledge, job autonomy, and access to organizational resources. Work experience and managerial position may also be related to role knowledge, decision latitude, and resource availability, although managerial roles may involve both resources and demands (32, 41). Gender was included because emotional labor and expectations for emotional display may differ across gendered work roles (14, 15).

Hypothesis 3. Demographic characteristics were expected to be associated with profile membership. Employees with higher education, longer work experience, and managerial positions were expected to be more likely to belong to adaptive profiles. By contrast, younger employees, women, employees with lower education, and non-managerial staff were expected to be more likely to belong to vulnerable or high-risk profiles.

## 3 Method

### 3.1 Study design, procedure, and methodological rationale

A cross-sectional questionnaire design was used because the study aimed to identify naturally occurring employee profiles and compare their concurrent associations with health and work outcomes. Cross-sectional designs are appropriate for describing patterns and associations at a single point in time, while their limits for temporal ordering and causal inference must be acknowledged (51). Accordingly, this design was suitable for the descriptive and person-centered purpose of the study. The aim was not to establish causal effects, but to map how emotion regulation burdens and psychological resources were configured within employees at one point in time.

Latent profile analysis was selected because it allows researchers to identify unobserved subgroups based on the joint distribution of multiple continuous indicators (45). This method is especially useful when the research question concerns configurations of risk and resources rather than the average association between one variable and another (46). In the present study, LPA allowed surface acting, deep acting, emotion regulation difficulties, resilience, self-efficacy, and optimism to be examined as a combined profile system.

Data were collected during voluntary workplace seminars on mental health and emotion management. Before each seminar, the presenter explained the study purpose, procedure, confidentiality arrangements, and voluntary nature of participation. Participants completed either a paper questionnaire or an online version accessed through a QR code. The first page included an informed consent statement describing the study purpose, potential risks and benefits, confidentiality, data use, and the right to refuse or withdraw. Only questionnaires with informed consent were retained. No personally identifying information was collected. Each questionnaire was assigned an ID number and stored in an encrypted file accessible only to the research team. The study was approved by the Institutional Review Board of Wuhan University (Approval No. WHU-HSS-IRB2025042).

### 3.2 Participants

Participants were employed staff members who attended the seminars and voluntarily completed the questionnaire. Inclusion criteria were: (a) at least 18 years of age, (b) full-time or stable part-time employment, and (c) ability to understand and independently complete a Chinese questionnaire. Exclusion criteria were implausibly short completion time, substantial missing data on key scales, or invalid response patterns such as selecting the same response option across the entire questionnaire.

A total of 1,700 questionnaires were distributed and returned across multiple seminars. After screening, 1,552 valid questionnaires remained, yielding a valid response rate of 91.3%. Age responses were checked for plausibility and formatting errors. Clear textual age entries were converted to numeric values, whereas implausible or unverifiable age responses were treated as missing for age-related analyses only. The full sample was retained for the main analyses, and valid age data were available for 1,542 participants. Sample characteristics are reported in **Table 1**.

**Table 1:** Participant characteristics.

Variable	Category	n	%
Gender	Male	645	41.6
	Female	907	58.4
Age (years)	M = 35.21, SD = 8.66, range = 20–62 (valid n = 1542)	1542	99.4
Education	Associate degree or below	298	19.2
	Bachelor’s degree	971	62.6
	Master’s degree	270	17.4
	Doctoral degree or above	13	0.8
Total work experience	< 5 years	374	24.1
	5–10 years	368	23.7
	11–15 years	382	24.6
	16–20 years	153	9.9
	≥ 21 years	275	17.7
Management tenure	< 3 years	983	63.3
	3–5 years	203	13.1
	6–10 years	166	10.7
	≥ 11 years	200	12.9
Management level	Senior management (e.g., CEO/general manager/vice president)	44	2.8
	Middle management (e.g., department head/division manager)	288	18.6
	Frontline management (e.g., supervisor/team leader)	468	30.2
	Non-managerial staff	752	48.5
Industry	Manufacturing	173	11.1
	Information technology/Internet	43	2.8
	Finance/Banking/Insurance	721	46.5
	Education/Research	31	2.0
	Medical/Health care	138	8.9
	Construction/Real estate	20	1.3
	Wholesale, retail, or trade	61	3.9
	Government agencies/Public institutions	128	8.2
	Consulting/Training/Service	87	5.6
	Transportation/Logistics	134	8.6
	Culture/Media/Advertising	16	1.0
Organization type	State-owned enterprise	1133	73.0
	Private enterprise	229	14.8
	Foreign-funded/Joint venture	20	1.3
	Government/Public institution	159	10.2
	Self-employed/Start-up	11	0.7

### 3.3 Measures

All variables were assessed with self-report questionnaires. Unless otherwise noted, higher scores indicate higher levels of the corresponding trait or symptom. Reliability coefficients (Cronbach’s α) for all scales are reported in **Appendix Table A1**.

#### 3.3.1 Depression

Depressive symptoms were measured with the Depression subscale of the Depression Anxiety Stress Scales 21 (DASS 21; 52). The DASS 21 contains 21 items and three 7 item subscales assessing depression, anxiety, and stress. The simplified Chinese version has shown good reliability and validity among Chinese college students (53). The present study used the 7 depression items (Items 3, 5, 10, 13, 16, 17, 21), which refer to depressive mood and anhedonia during the past week. Responses were originally rated from 0 (did not apply to me at all) to 3 (applied to me very much or most of the time) and were recoded to range from 1 to 4 by adding 1 to each item before analysis. The mean score of the seven items was calculated, with higher scores indicating more severe depressive symptoms. Internal consistency in this sample was α = .84.

### 3.3.2 Difficulties in emotion regulation

Difficulties in emotion regulation were assessed with the 18 item Difficulties in Emotion Regulation Scale (DERS 18; 54), derived from the original DERS (22). The DERS 18 covers six dimensions: nonacceptance of emotional responses, difficulties engaging in goal directed behavior, impulse control difficulties, lack of emotional awareness, limited access to emotion regulation strategies, and lack of emotional clarity. The Chinese version uses a 5 point scale from 1 (almost never) to 5 (almost always), with some items reverse coded. After reverse coding the relevant items, the mean score across the 18 items was calculated, with higher scores indicating greater difficulties in emotion regulation. The mean score was used in the analyses. Internal consistency was α = .86.

#### 3.3.3 Emotional labor

Emotional labor was measured with an 11 item scale commonly used in Chinese employee samples, based on the multidimensional structure proposed by Brotheridge and Lee (55) and adapted by Liu (56). The scale differentiates deep acting and surface acting. Items 1 to 7 assess surface acting, that is, suppressing, faking, or modifying outward emotional expressions without changing the underlying feelings. Items 8 to 11 assess deep acting, that is, efforts to genuinely experience the emotions required at work. Responses were given on a 5 point scale from 1 (almost never) to 5 (almost always). Mean scores were computed separately for deep acting and surface acting, with higher scores indicating more frequent use of the corresponding strategy. Internal consistency was α = .84 for surface acting and α = .75 for deep acting.

#### 3.3.4 Psychological resilience

Psychological resilience was assessed with the 10 item Connor-Davidson Resilience Scale (CD-RISC-10; 26, 57). The CD-RISC-10 was derived from the original 25 item scale and captures the ability to recover and maintain functioning in the face of adversity. The Chinese version used here employs a 5 point scale from 0 (never) to 4 (almost always). Items were originally rated on a five-point scale ranging from 0 (never) to 4 (almost always) and were recoded to range from 1 to 5 by adding 1 to each item before analysis. The mean score of the 10 items was calculated, with higher scores indicating greater resilience. Internal consistency in this sample was α = .93.

#### 3.3.5 Psychological capital: self-efficacy and optimism

Psychological capital was measured with the self-efficacy and optimism subscales of the Psychological Capital Questionnaire-24 (PCQ-24; 27, 28). The PCQ-24 includes four six-item components: self-efficacy, hope, resilience, and optimism. Because resilience was assessed separately with the CD-RISC-10 and the present study focused on core positive resources relevant to workplace adaptation, only the self-efficacy and optimism subscales were used. Items were rated on a five-point scale from 1 (strongly disagree) to 5 (strongly agree). Mean scores were calculated for each subscale, with higher scores indicating higher self-efficacy and optimism. Internal consistency was α = .88 for self-efficacy and α = .64 for optimism.

#### 3.3.6 Insomnia

Insomnia symptoms were assessed with the Athens Insomnia Scale (AIS; 58). The AIS has 8 items assessing sleep onset, night awakenings, early morning awakening, total sleep time, subjective sleep quality, daytime mood, daytime functioning, and daytime sleepiness. Each item was originally rated from 0 (no problem) to 3 (serious problem) and was recoded to range from 1 to 4 by adding 1 to each item before analysis. The eight recoded item scores were summed to yield a total score ranging from 8 to 32, with higher scores indicating more severe insomnia symptoms. A Chinese version that has been widely applied in Chinese samples was used (59). Internal consistency for the total score was α = .85.

#### 3.3.7 Psychological well-being

Psychological well-being was measured with a revised Chinese version of Ryff’s Psychological Well-Being Scales (36, 38, 60). The scale assesses positive psychological functioning in domains such as self-acceptance, positive relations, personal growth, purpose in life, environmental mastery, and autonomy. The Chinese version uses a 6 point response format from 1 (strongly agree) to 6 (strongly disagree), with some items reverse coded. After reverse coding the relevant items, the mean score across the 20 items was calculated, with higher scores reflecting greater psychological well-being. Internal consistency in this sample was α = .873.

#### 3.3.8 Work engagement

Work engagement was assessed with the 9 item Utrecht Work Engagement Scale (UWES 9; 37). The UWES 9 includes three dimensions, vigor, dedication, and absorption, each represented by three items. Items were originally rated on a seven-point scale ranging from 0 (never) to 6 (always) and were recoded to range from 1 to 7 by adding 1 to each item before analysis. The mean score of the nine items was used as an overall index of work engagement, with higher scores indicating greater work engagement. Internal consistency for the total score was α = .94.

#### 3.3.9 Innovative work behavior

Innovative work behavior was measured with the Innovative Work Behavior scale (IWB; 39). The Chinese version contains 10 items assessing idea exploration, idea generation, idea championing, and idea implementation in daily work, such as noticing problems beyond routine tasks, proposing original solutions, persuading others to support new ideas, and integrating ideas into work processes. Items were rated on a 5 point scale from 1 (never) to 5 (always). The mean of all items was used as an overall indicator of innovative work behavior, with higher scores reflecting more frequent innovation related behaviors. Internal consistency for the total score was α = .93.

### 3.4 Data analysis

Analyses were conducted in R 4.3.2 using tidyverse, psych, tidyLPA, lavaan, nnet, mlogit, and VGAM. Two-sided tests with α = .05 were used. After data screening, scale scores were calculated for depression, emotion regulation difficulties, surface acting, deep acting, resilience, self-efficacy, optimism, insomnia, psychological well-being, work engagement, and innovative work behavior. Reliability, descriptive statistics, and Pearson correlations were first examined.

Latent profile analysis was then conducted using six continuous profile indicators: surface acting, deep acting, difficulties in emotion regulation, resilience, self-efficacy, and optimism. LPA was appropriate because the first research question concerned whether employees could be grouped into qualitatively meaningful configurations based on multiple burdens and resources. Two- to five-profile models were estimated.

Model selection was based on information criteria, entropy, class sizes, posterior classification probabilities, elbow plots, and theoretical interpretability, following recommendations for latent class and latent profile model selection (45). Participants were assigned to the profile for which they had the highest posterior probability.

Profile differences in depression, insomnia, psychological well-being, work engagement, and innovative work behavior were tested with one-way ANOVAs and Tukey *post hoc* comparisons. This analysis addressed the second research question by comparing health and work outcomes across profiles. Finally, multinomial logistic regression examined demographic correlates of profile membership because the third research question concerned associations between demographic characteristics and a nominal four-category profile outcome. Odds ratios, 95% confidence intervals, and p values were reported.

## 4 Results

### 4.1 Sample characteristics

A total of 1,552 employees were included in the analyses (**Table 1**). Valid age information was available for 1,542 participants after age screening; these participants ranged from 20 to 62 years old (M = 35.21, SD = 8.66). The sample was slightly more female than male and included employees from different job levels, tenure groups, industries, and organization types. Most participants held at least a bachelor’s degree, and nearly half were non-managerial staff.

### 4.2 Descriptive statistics and correlations

Means, standard deviations, and Pearson correlations are reported in **Table 2**. Surface acting and deep acting were both slightly above the scale midpoint and were weakly positively correlated. Difficulties in emotion regulation were moderately positively associated with surface acting but were not associated with deep acting, suggesting that dysregulation was more closely linked to response-focused emotional display than to deeper emotional adjustment.

**Table 2:** Descriptive statistics and correlations among study variables.

No.	Variable	M	SD	1	2	3	4	5	6	7	8	9	10	11
1	Surface	3.47	0.66	—										
2	Deep	3.53	0.68	.14***	—									
3	DERS	2.08	0.53	.28***	−.01	—								
4	Resilience	3.57	0.7	−.07**	.21***	−.49***	—							
5	Self-efficacy	3.68	0.66	−.01	.25***	−.35***	.65***	—						
6	Optimism	3.48	0.54	−.20***	.20***	−.43***	.60***	.55***	—					
7	Depression	1.61	0.52	.28***	−.08***	.57***	−.47***	−.38***	−.53***	—				
8	Insomnia	14.96	4.62	.25***	−.06*	.45***	−.31***	−.26***	−.36***	.52***	—			
9	PWB	4.16	0.65	−.28***	.09***	−.58***	.57***	.50***	.60***	−.57***	−.45***	—		
10	Engagement	3.83	1.1	−.16***	.27***	−.29***	.60***	.58***	.54***	−.46***	−.30***	.44***	—	
11	IWB	3.15	0.72	0.01	.27***	−.24***	.55***	.64***	.40***	−.29***	−.16***	.37***	.62***	—

DERS, difficulties in emotion regulation; PWB, psychological well-being; IWB, innovative work behavior. *p <.05, **p <.01, ***p <.001.

The three positive resource indicators--resilience, self-efficacy, and optimism--were strongly and positively interrelated. Difficulties in emotion regulation and surface acting were positively associated with depression and insomnia. They were negatively associated with psychological well-being and work engagement. Deep acting showed small or nonsignificant associations with negative outcomes, but it was positively related to resilience, self-efficacy, optimism, work engagement, and innovative work behavior.

Overall, the correlation pattern supported the theoretical expectation that emotion regulation burdens are linked to poorer health and work outcomes, whereas psychological resources are linked to more favorable adjustment. These results provided preliminary support for testing person-centered configurations of risk and resources.

### 4.3 Latent profile identification

**Figure 1:**
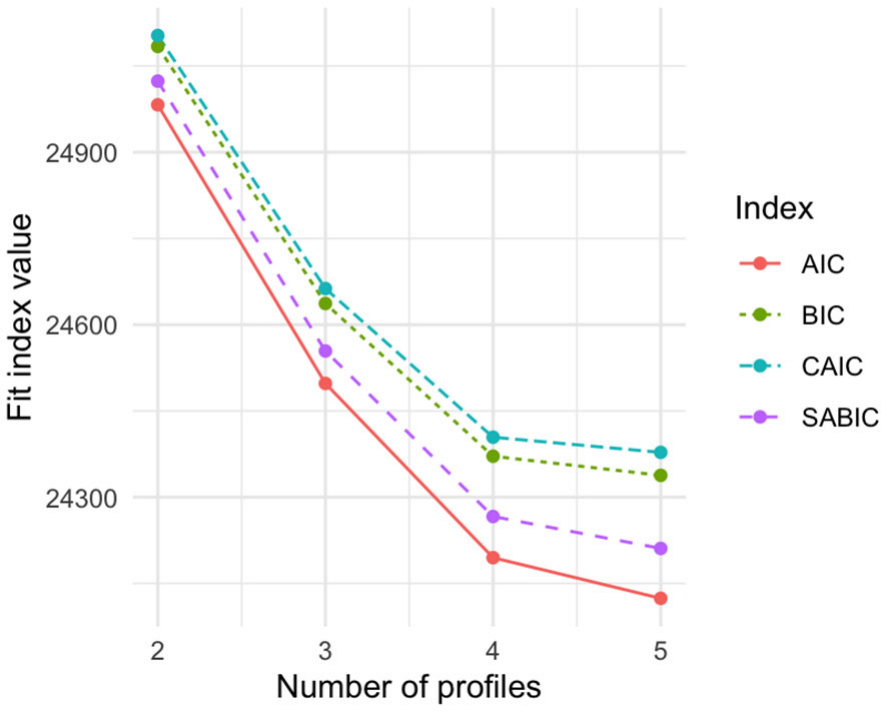
Fit indices for two- to five-profile latent profile models (N = 1,552).

Latent profile models with two to five profiles were estimated using the six profile indicators. Fit statistics are presented in **Appendix Table A2** and visualized in **Figure 1**. AIC, BIC, CAIC, and SABIC decreased substantially from the two-profile to the four-profile solution, and entropy improved from.78 to.83. The five-profile solution produced only small additional decreases in information criteria while increasing model complexity. Considering model fit, parsimony, class size, and theoretical interpretability, the four-profile solution was selected as the final model.

Under the four profile solution, each participant was assigned to the class with the highest Bayesian posterior probability. The profile sizes were as follows: vulnerable low-resource (n = 430, 27.7 percent), high-resource adaptive (n = 181, 11.7 percent), high-risk dysregulation (n = 57, 3.7 percent), and average (n = 884, 56.9 percent). As shown in **Appendix Table A4**, mean posterior classification probabilities for all four profiles were above.89, indicating good separation and classification accuracy.

**Figure 2:**
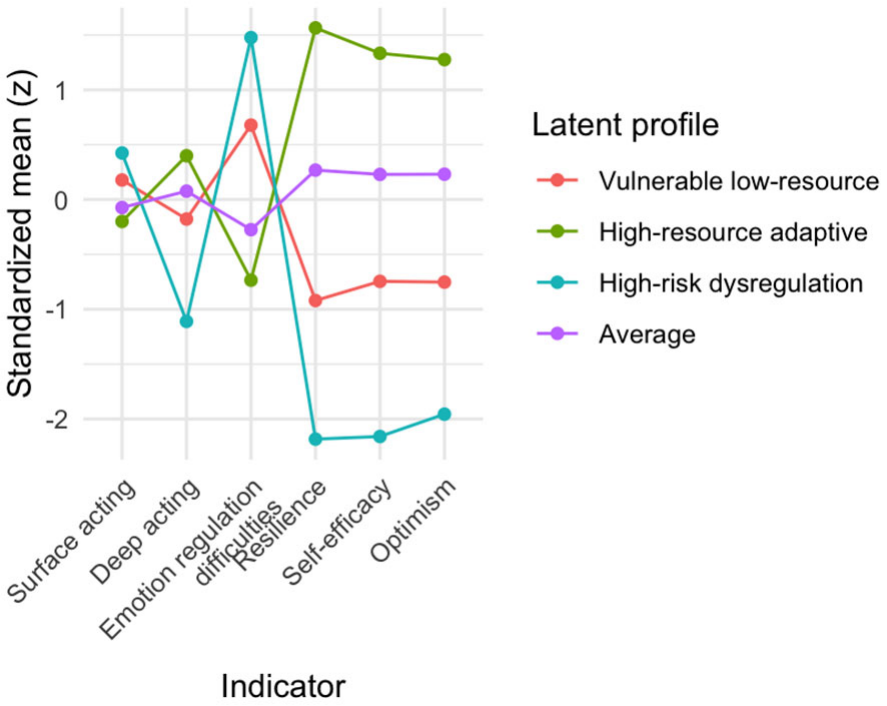
Standardized means of profile indicators across the four latent profiles.

**Figure 2** presents standardized means on the six indicators for each profile. The vulnerable low-resource profile showed higher difficulties in emotion regulation, slightly higher surface acting, slightly lower deep acting, and below-average resilience, self-efficacy, and optimism. The high-resource adaptive profile had the lowest difficulties in emotion regulation, the highest resilience, self-efficacy, and optimism, lower surface acting, and higher deep acting. The high-risk dysregulation profile showed the highest difficulties in emotion regulation, the lowest psychological resources, relatively high surface acting, and low deep acting. The average profile had z scores close to zero on all indicators and represented a normative moderate group. Taken together, the four-profile solution supported Hypothesis 1 because the expected high-resource adaptive, high-risk dysregulated, vulnerable low-resource, and moderate profiles were all identified.

**Figure 3:**
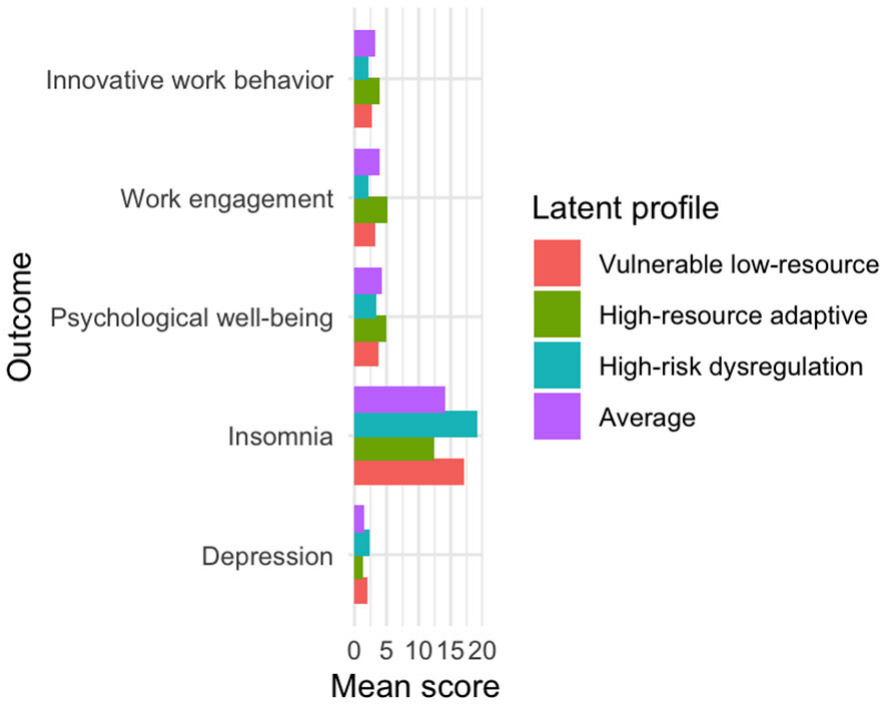
Mean levels of depression, insomnia, psychological well-being, work engagement, and innovative work behavior across the four latent profiles.

One-way ANOVAs were conducted to examine profile differences in mental health and work outcomes. Latent profile membership was the independent variable. Depression, insomnia, psychological well-being, work engagement, and innovative work behavior were the dependent variables. Profile-specific means are shown in **Appendix Table A3**, and the overall pattern is illustrated in **Figure 3**.

For negative outcomes, the high-risk dysregulation profile showed the highest depression and insomnia. The vulnerable low-resource profile followed, while the high-resource adaptive profile had the lowest scores. For positive outcomes, the high-resource adaptive profile showed the highest psychological well-being, work engagement, and innovative work behavior. The average profile was in the middle, and the vulnerable low-resource and high-risk dysregulation profiles showed progressively lower scores. Overall, the profiles formed a clear resource-risk gradient. This pattern supported Hypothesis 2 because the high-resource adaptive profile showed the most favorable outcome pattern and the high-risk dysregulation profile showed the least favorable pattern.

### 4.4 Demographic correlates of profile membership

Multinomial logistic regression examined demographic correlates of latent profile membership, with the Average profile as the reference group (**Table 3**).

**Table 3:** Multinomial logistic regression results for demographic correlates of latent profile membership (reference profile = Average).

Profile	Correlate	B	SE	p	OR	CI_low	CI_high
HRA	Age	0.019	0.020	0.346	1.02	0.98	1.06
HRA	Bachelor	0.523	0.255	0.040	1.69	1.02	2.78
HRA	Master	0.558	0.296	0.059	1.75	0.98	3.12
HRA	Doctorate	1.686	0.713	0.018	5.40	1.33	21.85
HRA	Female	0.177	0.169	0.296	1.19	0.86	1.66
HRA	Manager	-1.733	0.268	<.001	0.18	0.10	0.30
HRA	Tenure	0.196	0.132	0.137	1.22	0.94	1.57
HRD	Age	0.051	0.022	0.017	1.05	1.01	1.10
HRD	Bachelor	-0.682	0.325	0.036	0.51	0.27	0.96
HRD	Master	-1.637	0.530	0.002	0.19	0.07	0.55
HRD	Doctorate	0.767	1.164	0.510	2.15	0.22	21.10
HRD	Female	1.272	0.349	<.001	3.57	1.80	7.07
HRD	Manager	-1.526	0.340	<.001	0.22	0.11	0.42
HRD	Tenure	-0.687	0.167	<.001	0.50	0.36	0.70
VLR	Age	-0.020	0.016	0.231	0.98	0.95	1.01
VLR	Bachelor	-0.454	0.153	0.003	0.64	0.47	0.86
VLR	Master	-0.932	0.207	<.001	0.39	0.26	0.59
VLR	Doctorate	0.054	0.754	0.942	1.06	0.24	4.62
VLR	Female	0.408	0.124	<.001	1.50	1.18	1.92
VLR	Manager	0.245	0.193	0.204	1.28	0.88	1.87
VLR	Tenure	-0.133	0.103	0.196	0.88	0.72	1.07

Reference profile = Average. HRA, high-resource adaptive; HRD, high-risk dysregulation; VLR, vulnerable low-resource. Gender was coded as 0 = male and 1 = female. Education was treated as a categorical variable with associate degree or below as the reference category. Tenure was coded from 1 (< 5 years) to 5 (>= 21 years). Managerial position was coded as 0 = non-manager and 1 = manager. OR, odds ratio; CI, confidence interval.

For the high-resource adaptive profile, age and tenure were non-significant. Higher education was associated with higher odds of membership, especially for employees with a bachelor’s degree (OR = 1.69, p = .040) or a doctorate or above (OR = 5.40, p = .018). Managers were less likely than non-managers to belong to this profile (OR = 0.18, p <.001).

For the high-risk dysregulation profile, higher age was associated with higher odds of membership (OR = 1.05, p = .017). Women were also more likely than men to belong to this profile (OR = 3.57, p <.001). Higher education, managerial position, and longer tenure were associated with lower odds of membership.

For the vulnerable low-resource profile, age and tenure were non-significant. Higher education was associated with lower odds of membership, whereas women were more likely than men to belong to this profile (OR = 1.50, p <.001). Managerial status was not significant.

Overall, higher education was associated with more adaptive profile membership. Women were more likely to be in low-resource and high-risk groups, while managers and longer-tenure employees were less likely to be in the high-risk dysregulated profile. These findings partially supported Hypothesis 3. The findings for education and gender were broadly consistent with expectations, whereas the associations involving managerial position, age, and total work experience were more complex and did not uniformly follow the hypothesized pattern.

## 5 Discussion

### 5.1 Latent configurations of emotion regulation burdens and resources

Research Question 1 asked what latent profiles of emotion regulation resources could be identified, and Hypothesis 1 expected at least four theoretically meaningful profiles. The four-profile solution supported this hypothesis and provides a clearer person-centered account of how emotion regulation burdens and psychological resources are organized within employees. Person-centered approaches emphasize that individuals are not merely located along a single high-low continuum, but may form qualitatively distinct subgroups with different internal configurations (45, 46). In this study, the high-risk dysregulated, vulnerable low-resource, normative moderate, and high-resource adaptive profiles were not simply low, medium, and high versions of the same general resource dimension. Instead, surface acting, deep acting, emotion regulation difficulties, resilience, self-efficacy, and optimism combined into distinct and interpretable patterns.

The contrast between the high-resource adaptive profile and the high-risk dysregulated profile can be interpreted through Conservation of Resources theory and the Job Demands-Resources model. Conservation of Resources theory proposes that stress is closely tied to threatened, lost, or insufficiently replenished resources (50). The Job Demands-Resources model further suggests that personal resources are associated with lower strain and stronger motivational outcomes such as work engagement (32, 41). In this study, employees in the high-resource adaptive profile combined lower emotion regulation difficulties with stronger resilience, self-efficacy, and optimism. This configuration is consistent with a resource-rich pattern that may be more compatible with recovery from emotional demands and sustained work functioning. By contrast, the high-risk dysregulated profile combined high emotion regulation difficulties, relatively high surface acting, and weak psychological resources. This profile contrasts with the adaptive profile and is consistent with a resource-poor configuration in which emotional demands, limited regulatory capacity, and low personal resources co-occur.

The vulnerable low-resource profile adds a more nuanced finding. This group was not defined by the most extreme emotional labor pattern, but by insufficient psychological resources combined with elevated emotion regulation difficulties. Previous studies have shown that emotion regulation difficulties are associated with depression, insomnia, and other psychological symptoms (8). In contrast, resilience and psychological capital are generally linked with lower psychological distress and stronger work engagement (9, 30). Therefore, the vulnerable low-resource profile suggests that employee risk may be related not only to high emotional demands, but also to the absence of protective resources. This distinction has practical significance. Employees in the high-risk dysregulated profile may require more immediate mental health support and emotion regulation intervention. Employees in the vulnerable low-resource profile may be suitable candidates for earlier resource-building interventions, such as resilience training, psychological capital development, and strengthened workplace support. The normative moderate profile is also meaningful because it provides a reference group for identifying more targeted intervention priorities.

### 5.2 Resource-risk gradients in mental health and work functioning

Research Question 2 asked how the profiles differed in depression, insomnia, psychological well-being, work engagement, and innovative work behavior. The outcome comparisons supported Hypothesis 2. A consistent resource-risk gradient emerged across both negative health outcomes and positive work outcomes. Employees in the high-risk dysregulated profile reported the highest depression and insomnia, whereas employees in the high-resource adaptive profile showed the most favorable pattern of psychological well-being, work engagement, and innovative work behavior. This pattern is consistent with prior evidence showing that surface acting and emotion regulation difficulties are associated with burnout, depression, and sleep problems (7, 8). It also aligns with research indicating that psychological capital and related personal resources are positively associated with engagement and performance (31, 42).

These findings extend variable-centered evidence by showing that the combination of burdens and resources within individuals is also important. Depression and insomnia can be understood as indicators of resource depletion and impaired recovery. Work engagement and innovative work behavior, in contrast, reflect active resource investment. Employees need energy, confidence, and psychological flexibility to remain absorbed in work and to generate or implement new ideas. The Job Demands-Resources model suggests that resources are associated not only with lower strain, but also with a motivational process related to engagement and performance (41, 61). Accordingly, the profiles were linked to both poorer health outcomes and more positive work functioning, which confirms the central expectation of Hypothesis 2.

### 5.3 Demographic patterning of profile membership

Research Question 3 asked whether gender, age, education, managerial position, and work experience were associated with profile membership. Hypothesis 3 was partially supported. Education was the most consistent demographic correlate of profile membership. Employees with higher education were more likely to belong to the high-resource adaptive profile and less likely to belong to vulnerable or high-risk profiles. This pattern may reflect differences in coping knowledge, problem-solving experience, job autonomy, or access to organizational support. Prior organizational research has shown that psychological capital, work engagement, and job performance are positively related (30, 44). Gender also differentiated profile membership. Women were more likely than men to belong to the vulnerable low-resource and high-risk dysregulated profiles. This pattern may reflect gendered emotional labor demands and unequal expectations regarding emotional display, interpersonal care, and relationship maintenance at work (14, 15). However, this interpretation should remain cautious because job type, family responsibilities, and gendered role expectations were not directly measured.

The findings for managerial position, tenure, and age were more complex and did not fully align with Hypothesis 3. Managers were less likely to belong to the high-risk dysregulated profile, suggesting that role experience, decision latitude, or access to organizational resources may be relevant to lower profile-related risk. This interpretation is consistent with the Job Demands-Resources model, which emphasizes the protective role of autonomy and resource availability (32, 41). At the same time, managerial status was not uniformly associated with membership in the most adaptive profile. This unexpected pattern may indicate that managerial responsibilities are related to both resources and demands. Longer tenure was associated with a lower likelihood of membership in the high-risk dysregulated profile, which may reflect accumulated coping experience or selection effects. The positive association between age and high-risk dysregulated membership contrasted with the original expectation that younger employees would be more likely to belong to risk profiles. This finding should be interpreted cautiously and may reflect accumulated strain among some older employees rather than a general developmental pattern.

## 6 Conclusions

The present study identified four emotion regulation resource profiles among Chinese employees: high-risk dysregulated, vulnerable low-resource, normative moderate, and high-resource adaptive. These profiles differed systematically in depression, insomnia, psychological well-being, work engagement, and innovative work behavior. The findings show that workplace emotion regulation risk is better understood as a configuration of emotional demands, regulation difficulties, and psychological resources than as the result of any single indicator alone.

The study has three main implications. Theoretically, it extends variable-centered research by demonstrating that emotional labor, regulation difficulties, and psychological resources operate as meaningful within-person configurations. It also refines the application of Conservation of Resources theory and the Job Demands-Resources model by showing how resource-rich and resource-poor patterns correspond to different mental health and work outcomes. Practically, profile information may help organizations design stratified support. Employees in high-risk profiles may be suitable candidates for emotion regulation training, sleep and mental health support, and referral pathways. Employees in vulnerable low-resource profiles may be suitable candidates for resilience and psychological capital interventions before more severe symptoms emerge. Methodologically, the study demonstrates the value of latent profile analysis for identifying actionable subgroups in workplace mental health research.

## 7 Limitations and future research

Several limitations should be considered when interpreting these findings. First, the cross-sectional design prevents causal conclusions. Although the high-risk dysregulated profile showed poorer mental health and work outcomes, it remains unclear whether weak emotion regulation resources preceded these outcomes or whether existing depression, insomnia, or low work functioning preceded lower employee resources. Future studies should use longitudinal designs, such as three-wave follow-ups, to examine prospective associations between profile membership and later changes in depression, insomnia, work engagement, and innovative work behavior. Longitudinal research could also examine whether employees move from high-risk or vulnerable profiles toward more adaptive profiles over time.

Second, all variables were measured using self-report questionnaires. This approach was appropriate for assessing subjective symptoms, perceived regulation difficulties, and psychological resources, but it may have inflated associations among variables. Future research should combine self-report data with other sources, such as supervisor ratings of work engagement or innovation, objective sleep indicators, sickness absence records, or performance evaluations. Such multi-source designs would provide stronger evidence for the practical meaning of the identified profiles.

Third, the sample was collected from workplace seminars and included a relatively high proportion of employees from state-owned and financial-sector organizations. Employees who attended mental health and emotion management seminars may differ from other employees in stress awareness or help-seeking motivation. Therefore, the four-profile structure should be replicated in more diverse occupational groups, such as service workers, healthcare employees, manufacturing workers, private-sector employees, and platform workers.

Fourth, psychological capital was represented by self-efficacy and optimism, whereas hope was not included and resilience was measured separately. Future studies could compare the present resource configuration model with a full psychological capital model. This would clarify whether employee profiles are mainly driven by broad psychological capital or by specific combinations of resilience, self-efficacy, optimism, and emotion regulation capacity.

Finally, future research should use experimental or quasi-experimental designs to examine whether these profiles are modifiable through intervention. For example, high-risk dysregulated employees may be suitable candidates for emotion regulation training, stress management, sleep support, and mental health referral. Vulnerable low-resource employees may be suitable candidates for resilience training, psychological capital development, and workplace support enhancement. Pre-test, post-test, and follow-up designs could examine whether employees shift from risk profiles to more adaptive profiles and whether such shifts are accompanied by better mental health and work outcomes.

## Data Availability

The datasets presented in this article are not readily available due to participant privacy. Requests to access the datasets should be directed to the corresponding author.
