# Supplementary material for: Emotion regulation resource profiles among employees: mental health and work outcomes
Source: Front Psychiatry. 2026 Jul 30;17:1873483. doi: 10.3389/fpsyt.2026.1873483 (PMC13470637; doi:10.3389/fpsyt.2026.1873483)
Supplement: Supplementary file 1 [file DataSheet1.docx]

**Appendix**

****Table A1. Reliability coefficients (Cronbach’s α) for study scales (N = 1,552)****

| Scale | Subscale | Items (k) | Cronbach’s α |
| --- | --- | --- | --- |
| DASS-21 Depression subscale | Depression symptoms | 7 | .836 |
| Difficulties in Emotion Regulation Scale–18 | Mean score | 18 | .859 |
| Emotional Labour Scale | Surface acting | 7 | .838 |
| Emotional Labour Scale | Deep acting | 4 | .745 |
| Connor–Davidson Resilience Scale–10 | Mean score | 10 | .928 |
| Psychological Capital Questionnaire | Self-efficacy | 6 | .884 |
| Psychological Capital Questionnaire | Optimism | 6 | .644 |
| Athens Insomnia Scale (AIS) | Total score | 8 | .846 |
| Psychological Well-Being Scale | Mean score | 20 | .873 |
| Utrecht Work Engagement Scale–9 | Mean score | 9 | .936 |
| Innovative Work Behavior Scale | Mean score | 10 | .926 |

****Table A2. Latent profile analysis model fit statistics (N = 1,552)****

| Class | BIC | SABIC | AIC | CAIC | Entropy | LogLik |
| --- | --- | --- | --- | --- | --- | --- |
| 2 | 25084.04 | 25023.69 | 24982.45 | 25103.04 | 0.78 | −12472.22 |
| 3 | 24636.87 | 24554.27 | 24497.84 | 24662.87 | 0.80 | −12222.92 |
| 4 | 24371.27 | 24266.44 | 24194.81 | 24404.27 | 0.83 | −12064.41 |
| 5 | 24337.95 | 24210.88 | 24124.06 | 24377.95 | 0.86 | −12022.03 |

Note. AIC = Akaike information criterion; BIC = Bayesian information criterion; SABIC = sample-adjusted BIC; CAIC = consistent AIC; LogLik = log-likelihood.

**Table A3. Means by latent profile (N = 1,552).**

| Profile | n | Dep. | Insom. | PWB | Work eng. | IWB |
| --- | --- | --- | --- | --- | --- | --- |
| VLR | 430 | 1.95 | 17.00 | 3.67 | 3.17 | 2.74 |
| HRA | 181 | 1.27 | 12.44 | 4.89 | 5.18 | 3.88 |
| HRD | 57 | 2.33 | 19.11 | 3.36 | 2.18 | 2.11 |
| Average | 884 | 1.46 | 14.22 | 4.31 | 3.98 | 3.27 |

Note. VLR = vulnerable low-resource; HRA = high-resource adaptive; HRD = high-risk dysregulation; PWB = psychological well-being; IWB = innovative work behavior.

**Table A4. Classification posterior probabilities for the four-profile solution.**

| Class | P1 | P2 | P3 | P4 |
| --- | --- | --- | --- | --- |
| 1 | **0.89** | 0.00 | 0.02 | 0.09 |
| 2 | 0.00 | **0.90** | 0.00 | 0.10 |
| 3 | 0.09 | 0.00 | **0.91** | 0.00 |
| 4 | 0.06 | 0.03 | 0.00 | **0.92** |
